# Integrating mental health into HIV prevention and care: a call to action

**DOI:** 10.1002/jia2.25748

**Published:** 2021-06-24

**Authors:** Robert H Remien, Vikram Patel, Dixon Chibanda, Melanie Amna Abas

**Affiliations:** ^1^ HIV Center for Clinical and Behavioral Studies and Division of Gender, Sexuality, and Health Department of Psychiatry NY State Psychiatric Institute and Columbia University New York NY USA; ^2^ Department of Global Health and Social Medicine Harvard Medical School Department of Global Health and Population Harvard TH Chan School of Public Health Boston MA USA; ^3^ University of Zimbabwe & London School of Hygiene & Tropical Medicine London UK; ^4^ Health Service and Population Research Department Institute of Psychiatry, Psychology Neuroscience King's College London London UK

**Keywords:** mental health, HIV prevention, HIV care, HIV treatment cascade, behavioural health, integrated care

Mental health is a universal human asset, indivisible from other public health priorities, and especially important to people living with and at risk for HIV [1, 2]. Much has been written about mental health and HIV in high‐resource settings, especially in the United States. However, 90% of all those living with HIV globally, and 83% of the world’s total population, live in low‐income and middle‐income countries (LMIC). Knowledge generated in high‐income countries may not translate to the very diverse contexts encountered in LMIC where factors such as poverty, patterns of the burden of disease, gender norms, discrimination against specific groups, and mental health resources vary greatly. Given this context, this Special Issue of the *Journal of the International AIDS Society* focuses on mental health and HIV, predominantly in LMIC settings with a high HIV burden, and among key populations at greatest risk of HIV, to highlight: (1) the linkage between mental health problems, and other psychosocial factors, which increase HIV risk; (2) the interventions and strategies to prevent HIV among people with mental health problems; (3) factors associated with common mental disorders among young people and adults living with HIV and (4) interventions to improve mental health among persons with HIV (PWH).

Papers in this Supplement related to HIV prevention focused on vulnerable groups at elevated risk for HIV acquisition. Collins *et al*., synthesized the literature on HIV prevention and mental health among key populations, including gay men and other men who have sex with men, female sex workers, transgender women, people who inject drugs and incarcerated people. This review highlights the relationship between mental health problems and HIV prevention outcomes, showing that symptoms of mental disorders and distress are associated with increased sexual risk behaviours and poor engagement with HIV prevention. The authors found that integrating a mental health component into a behavioural change intervention, or linking mental health services to combination prevention activities, significantly reduced risk behaviour and mental distress, and improved access to mental healthcare [3]. Velloza *et al*., studied mental health and use of pre‐exposure prophylaxis (PrEP) in adolescent girls and young women in Zimbabwe and South Africa, a population at high risk of HIV acquisition. They found that depressive symptoms were common and persistent, particularly in the context of engagement in transactional sex, inter‐personal violence and traumatic stress symptoms [4]. Notably, all these factors were associated with worse adherence to PrEP. Among female sex workers in Kenya, Leis *et al.,* observed elevated levels of depression and anxiety, and the strong association between these symptoms and reported interpersonal violence. They also found that recent client‐perpetrated emotional violence and increased years in sex work were associated with decreased PrEP use [5].

Two papers in this Supplement examined the burden and impact of mental health problems among PWH. Too *et al*., conducted a systematic review on the prevalence and correlates of common mental disorders among young people living with HIV in sub‐Saharan Africa. They report that there was an increased risk of common mental disorders, especially depression, in youth living with HIV, compared to their peers who were not living with HIV. They also highlight how bullying, HIV‐related stigma, lack of social support and poor adherence to antiretroviral therapy (ART) were associated with depressive symptoms [6]. Nguyen *et al*., found that depression and anxiety symptoms were associated with sub‐optimal adherence to ART among PWH in Vietnam and that hazardous alcohol use and alcohol dependence interacted with anxiety symptoms to exacerbate poor adherence to ART [7].

This Supplement includes two reviews on the effectiveness of mental health interventions in PWH. Bhana *et al*., carried out a narrative review of mental health interventions for young adults living with and affected by HIV [8]. They identified some promising approaches while concluding that there is a paucity of evidence on effective approaches and on understanding the corresponding mechanisms of change for this population. The diversity of existing interventions – many of which are not specific – make it difficult to discern best practice. They suggest a need to test simple, brief transdiagnostic interventions which are likely to be feasible in low resource settings by utilizing, for example lay counsellors who are supported by digital technology. Nakimuli‐Mpungu *et al*., conducted a systematic review on mental health interventions for PWH [9]. They found that psychological interventions with three or more “active ingredients” (e.g. positive coping skills, social support, sharing personal problems, behaviour activation and cognitive re‐structuring) are likely to be more effective than interventions with fewer ingredients; and that people are also more likely to adhere to interventions tailored to the cultural context. They found that studies testing the implementation of pharmacological treatments for mental disorders in PWH had been compromised by poor adherence (e.g. to antidepressant medication), and by “stock‐outs” of basic psychotropic drugs. These authors call for large‐scale definitive trials providing long‐term follow‐up data, including among PWH with severe mental disorders.

Also in this Supplement, Senn *et al*., provide us with a Viewpoint perspective highlighting three research gaps in the study of mental health among PWH: (a) understanding the complex interactions between biological, psychological, social and structural factors that influence mental disorders in PWH; (b) developing and testing interventions to address mental health, as well as co‐occurring psychological, social and structural factors, to improve HIV outcomes and (c) implementation science to understand how to best implement and scale‐up efficacious interventions to improve mental health and HIV outcomes [10]. Among the gaps in knowledge is how to improve both mental health and HIV outcomes in low‐resource settings [11]. In the United States, combined interventions which address health behaviours (e.g. addressing barriers to medication adherence) and also mental health serve to enhance physical health, mental health and overall well‐being [12, 13]. In this issue, Magidson *et al*., report on a pilot trial of “*Khanya*,” a task‐shared, peer‐delivered behavioural intervention in South Africa [14]. *Khanya* blends ingredients to improve ART adherence with those to address alcohol and substance use. They studied implementation outcomes and found that *Khanya* was feasible, acceptable, appropriate and delivered with fidelity by peers, suggesting that peers may be a potential strategy to extend task‐sharing models for behavioural health.

The research described in the series of papers in this Special Issue emphasizes the importance of integrating mental health care within the full range of HIV programmes, from those which seek to reduce risks for acquiring HIV in vulnerable populations to those which seek to improve mental health outcomes and HIV outcomes in persons who are receiving ART. Drawing on this evidence, and the evidence synthesized in a series of recent global mental health and reports from the World Health Organization (WHO) [15, 16, 17], we recommend a number of key strategies.

First, interventions to promote mental health literacy should be made available for everyone involved in HIV prevention and care in low‐resource settings. This should include those directly affected and their families, as well as informal and formal service providers, so that all key stakeholders are aware of the importance of mental health for HIV risk, and treatment and care. Second, we endorse WHO advice to integrate screening and treatment of depression into the HIV care cascade in prevention and healthcare settings, including community‐based practices [18]. For those with depression and also poor adherence to HIV medication, we endorse the integration of evidence‐based treatment for depression with evidence‐based adherence counselling [19, 20]. We recommend that mental health interventions be provided for those with a range of vulnerabilities and distress experiences, and not be restricted only to those with narrowly defined and diagnosed mental illness. Third, prevention must target social determinants across the life course, particularly in adolescence where risk factors can be similar to those in adults, but even more pronounced in terms of their negative consequences. These include experiences of trauma and violence and poor educational attainment, which are associated with both poor mental health and HIV health outcomes. Fourth, interventions should follow the principles of stepped care, with access to self‐help and simple, brief, transdiagnostic evidence‐based interventions for all, with a pathway in place to offer more intensive care for those with severe mental illness [21]. Fifth, programmes must leverage the resources available in the community, including deploying the strategy of peer support and existing HIV health workers, which have clearly defined roles in the HIV sector. In addition, because of the exponential rise in the use of mobile devices in LMIC there is a need to leverage digital technology particularly mobile phone‐based apps to improve reach, fidelity and efficient data collection. Sixth, there needs to be a focus not just on access to care but also its quality, in particular for psychosocial interventions which are proven to be highly effective when delivered by non‐specialists such as lay counsellors and community‐based outreach workers. Finally, we need to emphasize social justice and equity to reduce disparities and inequities, for example as experienced by sexual, gender, racial and ethnic minorities. Fairness must also be applied to the principles of building research capacity in LMIC [22, 23]. We hope for, and look forward to seeing more research on mental health and HIV led by researchers based in LMIC [24].

In our collective goal of taking interventions to scale, it is critical that models are developed from the outset with a focus on feasibility and long‐term sustainability. A key guiding principle is to combine mental health into HIV programmes, rather than siloing into specialist mental health services, which, apart from being scarce in most countries, are also associated with stigma and low levels of acceptability. A way to do this would be to utilize key points of the routine HIV care cascade for mental health integration, such as HIV testing, PrEP and ART initiation, and viral load testing, rather than requiring separate appointments and/or at different locations. Research is needed to learn about barriers and enablers of such integration, and about cost‐effectiveness of interventions which can be delivered by trained and supervised non‐specialists.

Unfortunately, recent progress in achieving global HIV targets has fallen behind, as a result of the COVID‐19 pandemic [25]. Furthermore, the health and psychosocial burden and inequities have not been shared equally within and between countries. Stigma and discrimination, along with other widespread inequalities, continue to serve as major barriers to ending AIDS, as well as addressing the COVID‐19 pandemic and mental health. As we strive to get back on track in combatting HIV and AIDS, we call upon HIV programmes to recognize that the aspiration of a world without HIV cannot be realized without integrating mental health across all HIV programmes and prevention and care settings. Investing in mental health pays dividends – for example every US$ 1 invested in expanding treatment for depression and anxiety leads to a return of US$ 4 in better health and capacity to work [26]. As decisions are made about spending money on HIV services, in the face of the COVID‐19 pandemic, we need to spend available funds wisely, guided by the best science and human rights. This calls for evidence‐informed mental healthcare to be at the core of the HIV response, and to embrace the diversity of experiences and strategies which work to improve the lives and well‐being of all citizens of the world.

## Competing interests

The authors have declared no conflict of interest.

## Authors’ contributions

All authors contributed to the conceptualization, writing and editing of this manuscript.
